# The Soundscape of the COVID-19 Lockdown: Barcelona Noise Monitoring Network Case Study †

**DOI:** 10.3390/ijerph18115799

**Published:** 2021-05-28

**Authors:** Daniel Bonet-Solà, Carme Martínez-Suquía, Rosa Ma Alsina-Pagès, Pau Bergadà

**Affiliations:** 1 Grup de Recerca en Tecnologies Mèdia, La Salle—URL, Quatre Camins, 30, 08022 Barcelona, Spain; daniel.bonet@salle.url.edu (D.B.-S.); carmenjulia.martinez@salle.url.edu (C.M.-S.); 2 Wavecontrol, c/Pallars, 65-71, 08018 Barcelona, Spain; pbergadac@gmail.com

**Keywords:** lockdown, soundscape, *L_Aeq_*, annoyance, WASN, Barcelona

## Abstract

The lockdown social measures in Spain due to COVID-19 caused a significant decrease in urban noise levels, which was observed in most of the large cities. This paper presents an analysis of the noise levels in Barcelona, Spain, by means of an accurate analysis of the most relevant sensors deployed in the Barcelona Noise Monitoring Network. In this work, we present the $L_{Aeq}$ levels in eight different locations from January 2020 to June 2020—from Superblocks to industrial zones—including and detailing all stages of the lockdown. Several comparisons were conducted with the monitoring data available from the former years (2019 and 2018—when available). The results of the analysis in Barcelona show a drastic $L_{Aeq}$ reduction (−9 dBA), especially in nightlife areas of the city, moderate to high $L_{Aeq}$ change (−7 dBA) in commercial and restaurants areas and a small decrease in $L_{Aeq}$ (−5 dBA) in dense traffic areas.

## 1. Introduction

Europe is currently undergoing a significant urbanization process, mainly due to economic reasons. However, severe downsides such as air, waste and noise pollution accompany the growth of population in a given area and the technological development necessary to provide several services. Noise pollution is reported to have a strong correlation and causality with several health problems [1], such as mental health issues, ischemic heart diseases and sleep disorders. Therefore, policy makers have issued some policies to assess and manage environmental noise [2].

Since the outbreak of COVID-19 at the end of 2019, health authorities imposed unprecedented containment measures, just after the WHO characterized COVID-19 as a pandemic, and provided guidance to flatten the contagion curve. The goal was to reduce the impact of COVID-19 on national healthcare systems and not only to properly treat COVID-19 patients but also to prevent the healthcare systems from collapsing. As a collateral effect of the lockdown, man-made noise was considerably diminished and hence many research activities have been carried out to take advantage of this situation and study the effects of lockdown on noise pollution. Since traffic volume was reduced to only essential activities and social events were banned, a reduction of noise pollution was expected in all cities and villages in Europe and around the world.

The literature about the effects of the COVID-19 lockdown on soundscape is wide and heterogeneous: from perception surveys, single-sensor monitoring noise in a European capital to huge acoustic sensor networks monitoring traffic noise, railway noise, airborne noise and human activity in a region of 10 million inhabitants. We first summarize the main surveys we have found in the literature about the perception of people regarding the levels of noise during the 2020 lockdown. We continue describing research studies based on little, non-permanent and medium sensor networks and we finally address big acoustic sensor networks deployed in big cities, whose outcomes and conclusions can be compared with those conclusions reached in this research work about the Barcelona soundscape during 2020 lockdown.

We first begin by highlighting a study on soundscape perception in Italy [3], which was developed by means of an 18-question survey distributed to residents (323 questionnaires analyzed) scattered in a wide area in Italy. The aim of the survey was to determine the change in soundscape perception of Italian people regarding the 2020 lockdown period. Results confirmed the expectation of a reduction of pollution noise. Furthermore, they correlated the survey outcomes with other variables, such as dwelling year of construction, number of flat mates in a dwelling, type of employment, etc., and they underlined the importance of evaluating a wide range of factors for a proper analysis. The results of this survey were intended to be useful for soundscape acousticians, citizen science and psychoacoustics experts as well as for environmental technicians and policy makers who can use this knowledge to understand potential benefits for the city and the environment. Another survey, which was launched online by *Centre d’Information sur le Bruit* and Bruitparif [4] between 18 March and 10 May, received 3242 valid questionnaires, from which 42% were residents of Île-de-France. Questionnaires were not random or quota-based but voluntary, hence their results cannot be statically representative but they can provide interesting indications about the feelings related to modifications of the sound environment during the 2020 confinement. Results of the survey showed that residents of Île-de-France felt a sharp decrease in the perceived intensity of noise in their homes, from all sources. In a scale of 0 to 10 the drop was 3.8 compared to the situation prior to the lockdown. Results showed that people who, on regular basis, were exposed to noise generated by activities (e.g., bars, shops, constructions sites, etc.) or airborne noise were those who noted the greatest change in sound intensity at their homes during the lockdown. The lockdown was accompanied by a decrease in the perception of sound sources: 92.3% of respondents noted a decrease in road traffic noise, 76.3% of respondents noted a decrease in airborne noise and 36.4% of respondents noted a decrease in railway noise. At the same time, an increase of the perception of sounds of natural origin was perceived by 85.4% of the respondents and 44.5% reported no change of sounds from neighbors. To sum up, the changes in the sound environment during confinement were overwhelmingly judged positive and perceived as pleasant, calm or peaceful by the respondents.

In the literature, measurements carried out by just one sensor placed in the center of a big city or measurements taken at several points during short periods of time can be found. We highlight the following ones placed at the city of Stockholm, at the tourist city of Granada, in the south of Spain, and at the historical neighborhood of Rio de Janeiro, Brazil.

The Swedish government opted for laxer mobility restrictions during the 2020 lockdown, while many European governments imposed severe constraints to personal and goods movement. This alternative approach to the pandemic was evaluated using an over 1 year noise recording campaign at one point in the city center of Stockholm, Sweden [5]. The sensor was placed at a crossroad between two major axes in the nearby bus and subway stations, as well as next to a leisure area full of bars and restaurants. The goal of this contribution was not to compare absolute data during lockdown with data prior to lockdown, but to compare fluctuations of noise coming from human activities as a response to the Swedish Public Health Agency announcing recommendations regarding social activity. Results showed that an average decrease of up to −2.9 dBA difference to a reference value on weekends occurred in the month of March and, with a shift of about a week, the weekdays showed similar drop values. From the month of April onward, a slow increase of measured values to the reference value was observed, stabilized on average around −1.0 dBA to the reference value, in June. The authors suggested that tighter self-imposed restrictions of the Swedish population might be the reason of the observed 1 dB lower level on weekend than on weekdays, computed as an average of the entire period of COVID-19 effects.

During the 2020 lockdown period in the city of Granada, Spain, a research work registered the sound pressure levels (SPL) at four typical tourist points with the aim to check the affect on soundscape due to the lack of human activity [6]. They compared 6 min of data gathered during lockdown with 6 min of data recorded 1 year before at the same points, with the intent to identify changes or shifts of temporal or spectral patterns. Results showed an astounding noise reduction of up to 30 dBA in $L_{Aeq}$, when comparing data from both years. They also showed LAF values distributions, with all four sites aggregated, and with lockdown values being considerably low compare to 2019 values. Finally, they concluded that spectral content suffered higher changes on sites where traffic noise was dominant.

Rio de Janeiro is a city in Brazil with 6.7 million inhabitants, which suffered the highest level of social isolation and quarantine during July 2020. During this time, some measurements of $L_{Aeq}$ were conducted at 12 different locations in the historical neighborhood of the city [7]. Data were gathered in an area of 776 m${}^{2}$ for 5 min at each location. Comparisons were made with data collected at the same points during 2019. They concluded that the largest noise reduction (i.e., between 10 dBA and 15 dBA) were observed at zones with low traffic flow but with heavy human activity (e.g., street sellers, loudspeakers, people walking, talking and speaking at cellphones). However, heavy traffic areas suffered a noise drop of only 3 dBA to 5 dBA, despite having a flow reduction of traffic of 52%.

Studies using a similar acoustic sensor network to the one used in this research, except not as broad and not taking into account the same number of noise sources, are developed in the following research.

An analysis of the noise levels in Girona [8], a city in the northeast of Catalonia, Spain with 100,000 inhabitants, showed the $L_{Aeq}$ levels in four different locations from January 2020 to June 2020, including all the stages of the lockdown. Several comparisons were conducted with the monitoring data available from the former years (2019, 2018 and 2017—when available). Sensor locations were chosen to study different types of noise sources (i.e., traffic, mixture of traffic and leisure, only leisure and a quiet zone in the old town). It concluded that there was a clear decrease of the noise in the street, coming from any of the urban noise sources. The sensors presenting larger differences between before and during the lockdown were those that contained night leisure noise, and they also presented larger differences with previous years (i.e., difference median values between 8 dBA and 12 dBA). The sensors with a heavy charge of traffic presented lower differences in all the conducted measurements (i.e., difference median values between 2 dBA and 8 dBA). The leisure noise values decreased especially during the evening and the night, and mainly in the leisure areas.

A study in the city of Buenos Aires [9], located in the Republic of Argentina, was performed using five acoustic sensors. The sensors were placed in significant artery roads where heavy traffic noise is produced. They compared the $L_{Aeq}$ at daytime, nighttime and the maximum permissible limit, which is established by a national law, with data recorded prior to the lockdown period. The comparison showed a decrease in gathered values during the lockdown period, which was even clearer at night when the maximum difference was of 6.9 dBA. However, the recorded values were not low enough to go below the maximum permissible level, even during the lockdown period—at only a couple of spots, the gathered values were lower than the limit. They concluded that only five sensors in a city with more than three million inhabitants might be too few and conclusions might not cover the acoustic behavior of the whole city during the 2020 lockdown.

Basu et al. [10] focused on the effects of COVID-19 lockdown in Dublin, Ireland, by means of twelve monitoring stations. They performed a regression-based trend analysis and a change point–point analysis on gathered data to investigate the changes in noise pollution and the point in time at which the observations changed. They concluded that a clear reduction in road and air traffic during the lockdown led to a reduction in noise pollution at all 12 stations. They also implied in the study that the reduction of traffic noise in cities is a good reason to promote feasible and alternative means of transport to improve public health.

The following research works are based on similar acoustic sensor networks and/or similar size and type of city compared to Barcelona. In some of these works, they also discriminate between several sources of noise and therefore their outcomes and conclusions could be compared to those reached in the current paper.

A quantitative study on noise levels by means of a 31-acoustic sensor network during lockdown and the following de-escalation periods was performed in the city of Madrid, Spain [11]. They grouped the sensors into three categories: traffic-dominated areas, city-center areas dominated by road and leisure noise and quiet residential areas. They compared lockdown data with data gathered prior to lockdown within 2020. They showed a clear decrease of the noise level, which reached a mean value of 7.4 dB in traffic dominated areas. They also observed a higher reduction of noise pollution on weekends rather than weekdays, which were attributed to the absence of commercial and leisure activity. Noise pollution reduction was noted to be higher at nights than during the day, which was due to the complete absence of leisure activity. These two phenomena were observed in road dominated and leisure areas but not in residential areas, where an exceptionally low reduction in noise level was reported. In this study, they also attempted to analyze the changes in time–noise patterns at the five analyzed points. They reported changes in the peak hours during the lockdown, as well as a different time span of noise activity.

Road traffic acoustic sensor networks were also used to monitor the reduction in noise pollution due to the drastic reduction of traffic volume during the 2020 lockdown period. The data provided by the 24 sensors, clustered in six groups by similar traffic features and located in the Milan municipality were averaged on weekly basis from March 2020 to May 2020 [12]. In the A90 highway encircling the city of Rome, Italy, three monitoring devices were used to retrieve data from the northeast area of the above-mentioned highway. In Milan, results showed an abrupt reduction of noise levels of both Lden and Ln during lockdown (from week 10 to week 18) and a slow return to previous values from May to June. The average difference compared to 2019 values, observed in the central part of the sensor area, is 7.3 dBA for Lden and 7.6 dBA for Ln. In Rome, a clear correlation in the decrease in light vehicle and heavy-duty vehicles by means of the traffic noise measured in A90 highway was shown, with an average reduction when compared to the 2019 data of 5.2 dBA of Lden and 5.4 dBA of Ln. In Milan, they also reported a decrease of up to 60% of the population exposed to high noise levels (i.e., *Lden* > 65 dBA), whereas for the Rome highway, the reduction was 63% during the day and 23% at night, when road traffic usually quietens.

A permanent sensor network deployed in Île-de-France made it possible to monitor the soundscape at many points in Paris and its surroundings, which has 10 million inhabitants, during the: 2020 lockdown weeks, previous and subsequent weeks [4]. It is a wide sensor network composed of the following dominant noise source stations: 18 road traffic noise stations, 9 railway noise stations, 18 airborne noise stations, 30 human activity noise stations in the eight inner districts of Paris and 17 construction noise stations. Stations dominated by traffic noise showed a mean reduction of 5.9 dBA regarding $L_{den}$ factor, with a more marked decrease on weekends than week days. A greater reduction of $L_{den}$ was observed at night (7 dBA) than during the day (4.6 dBA), as well as a higher reduction in Paris streets (7 dBA) than in surrounding highways (4.8 dBA). The deepest drop was observed between the 2nd and 6th week of confinement, with a rise in levels during the two last weeks of confinement and a final return to a close-to-normal situation since mid-June. Railway noise suffered a mean decrease of 5.3 dBA regarding the $L_{den}$ factor. Greater reductions were observed at night (6.6 dBA) than during the day (4.4 dBA). The most significant reductions were observed between the 2nd and 7th week of confinement with a slight rise of levels during the last week of confinement and a return to a close-to-normal situation since mid-June. Air traffic suffered a significant slowdown, between 85% and 90%, in flights during containment, with the exception of air cargo. A drastic reduction of airborne noise was measured, reaching up to 30 dBA in during specific weeks of confinement, around the main airports of Paris (i.e., Paris-Orly and Paris-CDG), which were closed during much of the confinement. Around aerodromes, a complete cessation of leisure flights meant a virtual disappearance of noise pollution; however, the noise pollution returned at significantly higher levels since early June. Regarding human activity in the lively quarters of the capital, strong reductions in sound level (between 6 dBA and 20 dBA) were observed. Different levels of noise reduction depended on the neighborhood, the type of day (week day of weekend) and the hour of the day, with maximum reduction at evening and early night in lively neighborhoods, which have many bars and restaurants or where public spaces are heavily used in appropriate weather. A fairly clear and gradual rise in noise levels accompanies the phases of confinement from a partial appropriation, the reopening of terraces and the full re-appropriation of public spaces. The shutdown of certain large construction sites during the lockdown resulted in reductions of ambient noise of up to 20 dBA. The return of noise pollution began between the end of April and the beginning of May a number of worksites returning to work.

The present contribution presents, for the first time, acoustic sound pressure levels gathered by means of an acoustic sensor network, composed of up to 70 sensors, placed in the city of Barcelona, Spain. It classifies and clusters the noise sources in a broad manner, i.e., it shows eight different noise sources and their evolution during the 2020 lockdown period as well as a comparison with two previous years. In Section 2, we describe the sensor network deployed in the city of Barcelona. In Section 3, we explain the lockdown stages that the population of Barcelona went through during 2020. In Section 4, we show the outcomes of our analysis (i.e., global city results and representative selected locations). Finally, in Section 5 we explain the conclusions we have reached.

## 2. Barcelona Noise Monitoring Network

Barcelona Noise Monitoring Network [13,14] is the name given to the network deployed in Barcelona in recent years. It consists of 112 devices, of which, 86 are sensors and 26 are sound level meters. In this work, we used data from 70 of the 86 sound sensors, which are deployed for long-term analysis in several pre-analyzed places in the city. Some of the sensors during the lockdown were not working properly, so their data were used for this analysis; only the data gathered from the sensors working under confidence conditions. The noise sources to be monitored by these sensors are mainly [14] road traffic noise, recreational noise (leisure noise), private activities noise, waste collection and construction works.

**Figure 1:**
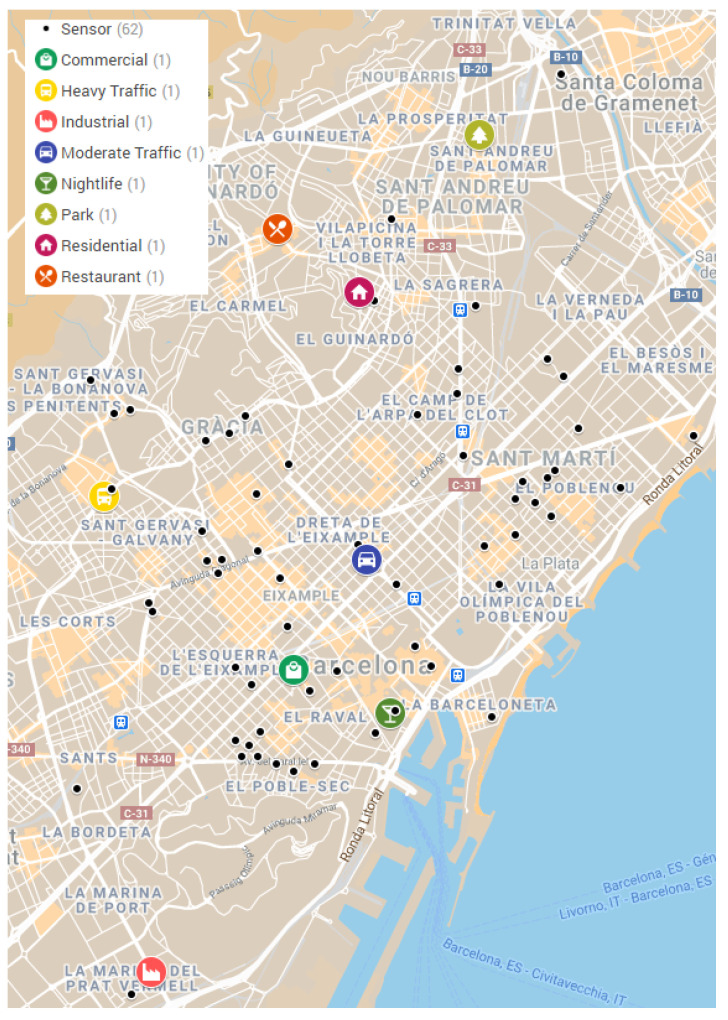
Map of the distribution of noise sensor devices of the Barcelona Noise Monitoring Network.

In Figure 1, we can observe the distribution of the noise sensors in the territory of Barcelona. Despite the fact that officials assign certain homogeneity between different districts [14], the precise measurements show that the city center (commerce and traffic) and the leisure areas have more devices deployed. The data used for this analysis were collected by these sensors, and in Section 4 we clarify the criteria used to choose some of them as the most representative of city noise changes, depending on the noise sources and the type of activity, during the 2020 lockdown.

**Figure 2:**
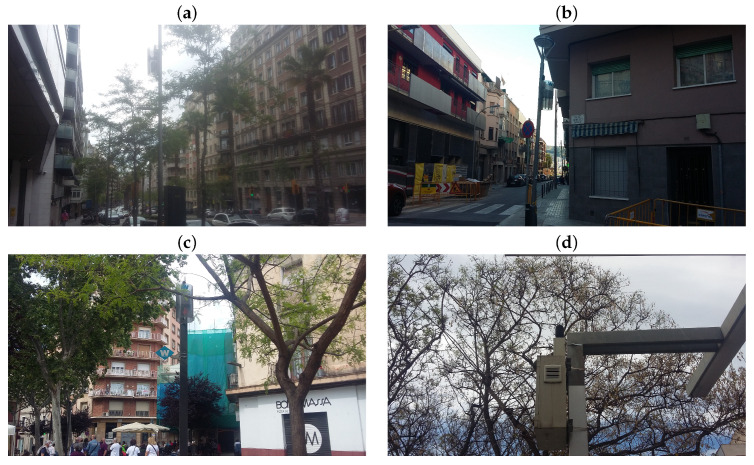
Selected sound level meters deployed in Barcelona: (**a**) General Mitre, 137. (**b**) Torre dels Pardals, 96. (**c**) Plaça Eivissa, 21. (**d**) Eiximenis, 21 (Jardins CASA BLOC).

Barcelona’s sound sensors network is integrated into the Sentilo BCN network of sensors [13] and actuators that collect and store information generated by Barcelona through a layer of sensors deployed across the city. The sensors used to gather the data used in the present paper are CESVA’s TA120 Class 1 sound level meters [15], which allow 24 h/7 d continuous measurement of $L_{Aeq}$ and offer protection against external agents. Furthermore, their light weight, small size, ease of integration into urban furniture and variable power and communication possibilities make them a perfect solution for outdoor noise monitoring in smart cities. Most of the sensors are attached to post lamps or similar urban structures at about 4 m above the floor level as seen in Figure 2 as is required in ISO1996-2 [16].

The data set obtained via this network is used in [17]. However, this present paper expands the analysis in Section 4 by means of giving an overview of the whole city through an aggregated study of the data gathered from all the available sensors, calculating an expanded array of noise indices, pointing out differences among days of the week and periods of the day by lockdown stage, exploring more kinds of noise sources beyond the traditional traffic and nightlife.

For the 2020 period, only 1.86% of the data were missing or anomalous, particularly from four specific days, i.e., 7 January 2020, 8 January 2020, 5 March 2020 and 19 May 2020. On the contrary, data coverage in prior years was not as thorough. For 2019, 10.83% of the data were missing. Nine out of seventy sensors had long periods of inactivity during the studied months and there were five series of days with a significant dip in the percentage of available data, i.e., from the 19th to 22nd of January, from the 2nd to the 4th of March, from the 3rd to the 8th of April, from the 1st to the 2nd of May and from the 20th to the 24th of May. In 2018, 11 of the 70 sensors were not yet deployed and 19 more were not operational during the whole semester. For that reason, the data coverage during 2018 was only about 71.3% of 2020’s network, where the first months of the year had less data coverage. However, aggregated data from both years increase the coverage to almost-2020 levels.

## 3. Barcelona Lockdown Stages

In order to best analyze the environmental sound in Barcelona during the COVID-19 pandemic compared to prior years, ten differentiated temporal stages have been defined according to the restrictions imposed on the city at any given time frame.

- Stage 1 (Pre-Lockdown): From 1 January 2020 to 13 March 2020. This period is free from any COVID-19 related restrictions and it should serve to run baseline comparisons with prior years.
- Stage 2 (Partial Lockdown 1): From 14 March 2020 to 29 March 2020. Starting 13 March 2020 [18], Catalan schools and universities were closed. In 14 March 2020 [19], an extraordinary meeting of the Council of Ministers decreed a state of emergency in Spain. During this period, activities were limited to basic needs, such as buying food or medication but people continue to work. Passenger transport, both public and private, was reduced.
- Stage 3 (Total Lockdown): From 30 March 2020 to 13 April 2020. Spanish government tightened the lockdown. A total lockdown was called and all non-essential workers were to remain at home until 9 April 2020, coinciding with Easter Holidays’ beginning [20].
- Stage 4 (Partial Lockdown 2): From 14 April 2020 to 25 April 2020. In spite of the state of emergency being extended [21], lockdown no longer applied to non-essential workers. Thus, restrictions were the same as in Stage 2.
- Stage 5 (Outdoor Activities): From 26 April 2020 to 3 May 2020. Children were allowed to go out for a walk in Catalonia, starting 26 April 2020 [22]. Six days later, adults were allowed to practice sports outdoors. Different time slots were assigned to different age groups [23].
- Stage 6 (Shops and Takeaway): From 4 May 2020 to 17 May 2020. Stage 6 marks the beginning of the de-escalation process [24]. During this first stage of de-escalation, people were allowed to go out during assigned time slots. Shops and other businesses could open but only by appointment. Bars and restaurants were open only for takeaway orders.
- Stage 7 (Museums and Sports): From 18 May 2020 to 24 May 2020. In this stage, shops could open without prior appointment. Museums and libraries could open. Professional sport could be started up again.
- Stage 8 (Bars and Restaurants): From 25 May 2020 to 7 June 2020. Bars and restaurants’ terraces could open at 50% capacity. Hotels could also open.
- Stage 9 (Entertainment and Mobility): From 8 June 2020 to 17 June 2020. Cinemas, theaters and shopping centers could open with reduced capacity. Mobility to second residences was allowed.
- Stage 10 (Back to Normality): From 18 June 2020 to 30 June 2020. During this final stage of the de-escalation process, nightclubs could open and almost all activities could take place at a higher level of capacity.

These ten stages can be grouped and reduced to three to perform a more general comparison between the pre-lockdown, lockdown and de-escalation time frames. For our study, Stage A refers to the pre-lockdown period, corresponding to Stage 1. Stage B refers to the lockdown period, corresponding to Stages 2 to 5. Finally, Stage C refers to the period when the Spanish de-escalation plan took place in Barcelona and corresponds to Stages 6 to 10.

## 4. Results

This section details the analysis conducted over the 70 sound sensors coming from the Barcelona Noise Monitoring Network. In the following subsections, several metrics are used to evaluate the noise performance in the city, both during the 2020 lockdown but also comparing these values to the former 2019 and 2018 records when available. First, a general approach to the city tendencies is conducted and, then, several sensors are chosen to analyze in detail, with special attention to the noise sources changes of each sensor during the lockdown.

### 4.1. Global Barcelona Results

This first section is devoted to several comparisons conducted for all the values gathered and available in the Wireless Acoustics Sensor Network in Barcelona; in this sense, the results presented do not correspond to any specific sensor or location in the city, but to an aggregated value of 70 samples distributed through the city, mainly in the city center.

#### 4.1.1. Environmental Noise in Barcelona 2018 to 2020 Comparison

**Figure 3:**
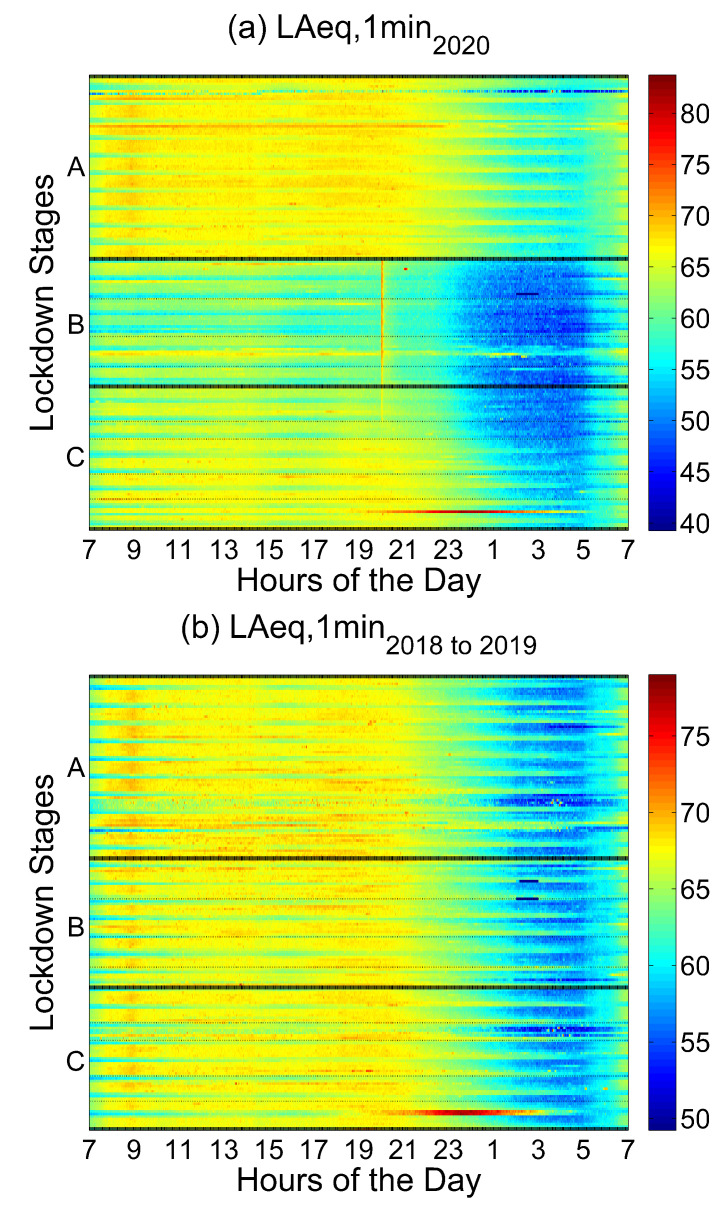
$L_{Aeq}$ Mean value of 70 Barcelona sound sensors: (**a**) global $L_{Aeq}$ mean value in Barcelona during the different lockdown stages in 2020; (**b**) global $L_{Aeq}$ mean value in Barcelona during the equivalent weeks in 2018 and 2019.

Figure 3 shows the comparison between the $L_{Aeq}$ gathered from January to June 2020 with the data collected during the same period of 2018 and 2019. In this comparison, data from former years were averaged and 70 sensors out of 86 were used.

An overall trend of environmental noise in Barcelona is depicted in Figure 3. The main goal is not to show a specific area of the city but an overview in terms of noise reduction regarding lockdown stages and hours of the day. Focusing on Stage B (Lockdown), there are considerable differences among its internal divisions. The second week of Stage 2 (Partial Lockdown 1) and Stage 3 (Total Lockdown) are the most significant periods in noise reduction terms as they correspond to the more restrictive weeks during the lockdown. Moreover, the higher reduction in noise levels appears at nights, from midnight to 5. This behavior is correlated with other cities’ trends, such as Madrid [11]. During the final weeks of Stage B and the first weeks of Stage C, corresponding to Stage 4 to 7, a slightly continuous decrease in terms of noise reduction against the same period during 2018 and 2019 (the average) is seen.

Similarly, in the final weeks of Stage C a continuous decrease in terms of noise reduction is also shown. This is due to the gradual opening of restaurants, terraces, cinemas and night clubs, among other activities. It has a clear impact on daytime noise levels, but especially on noise levels during the night period.

In addition, an anomalous event can be seen on 23rd of June (Stage C) in Figure 3a,b from 19 to 2. This coincides with Saint John’s Eve celebration, an important night where bonfires are made and firecrackers are thrown. Furthermore, another important event can be identified in Figure 3a. During Stage B and the first weeks of Stage C (Stages 2 to 7), an increase in the noise level is detected at 20 o’clock. This singularity corresponds with the popular applause to healthcare professionals carried out from windows and balconies throughout the city.

In Table 1, statistical noise levels (LAN) calculated for the first semester of 2020 against the same period during 2018, 2019 and the average of 2018 and 2019 are shown. LAN are used to better understand noise level fluctuations over a period of time. For example, LA90 describes the level (A-weighted) that was exceeded 90% of the time, whereas LA10 describes the level that was exceeded for only 1% of the time. In addition, variation between LA10 and LA90 was calculated to express variability in terms of noise levels. LA10–90 index shows the differences regarding LA10 and LA90 statistical noise indices, during the first semester of 2018, 2019 and 2020. Furthermore, average $L_{Aeq}$ noise levels are presented for the same period. All these noise indices were calculated from the aggregated data gathered with the same 70 sensors, as shown in Figure 3.

Looking at $L_{Aeq}$ values in Table 1, noise reduction between the same period in 2020 to 2018–2019 is −2.5 dB on average. Interestingly, the average noise level reduction between 2020 and 2019 is lower (−1.8 dB). Comparing each consecutive year’s variation, a progressive noise reduction trend through the past three years can be noticed. It is remarkable that 2019, a year free of pandemic-related restrictions, already experienced a substantial reduction in the first semester’s average $L_{Aeq}$ compared to 2018.

The percentiles in Table 1 show a higher variability of noise levels during lockdown—this is due to an increase in level range between the lowest and the highest measured level (see Figure 3). In particular, the difference between *LA*90 and *LA*10 during the same period of 2020 to 2018–2019, on average, is 4.9 dB. It is almost 1 dB and 3 dB higher than the difference between 2020 to 2019 and 2019 to 2018 accordingly.

#### 4.1.2. Average Environmental Noise by Lockdown Stage

In Table 2, $L_{Aeq}$ from the different stages during the first semester of 2018, 2019 and 2020 (lockdown) are shown. Furthermore, differences between years are computed in order to evaluate the incidence of lockdown in average levels.

In Stage A, which corresponds with the pre-lockdown period, the most significant differences are between 2018 to 2019. In contrast, for Stage B and Stage C, the most meaningful differences are shown in the 2020 to 2018–2019 column of Table 2. Differences between 2020 and the average of 2019 and 2018 during Stage B are about −6 dB. As was to be expected, the higher difference takes place during Stage 3 where the total lockdown was imposed. Interestingly, even though restrictions during Stage 2 were basically the same as in Stage 4, there is a higher level of noise during the latter period—this correlates with a change in the behavior of Barcelona’s inhabitants after the Easter holiday, where a lower number of people stayed at home compared to the first partial lockdown stage, according to a study conducted by Satya Insights [25]. This is due to the improvement of the pandemic’s indices and the exhaustion of homebound citizens.

In Stage C, the average variation for the same years is almost −3 dB. However, looking into the detailed stages of the de-escalation process, there is a marked difference between Stages 6 and 7 and the final three stages. In Barcelona and other densely populated cities, people were more worried about leaving the COVID-free safety of their homes during these initial weeks compared to smaller municipalities [26]. On the one hand, as the main new activities allowed from Stage 6 were mainly shops and takeaway restaurants, many people preferred to keep shopping online and eating at home [27]. On the other hand, the opening of bars and restaurants during Stage 8 had a clearer effect on social interaction, which translated to higher noise levels.

Furthermore, the highest noise level was measured in 2018 during Stage A (66.4 dBA) and the lowest in 2020 during Stage B (59.5 dB). This noise level was expected if restrictions in Stage B are considered. The changes in the daily life of Barcelona’s inhabitants show a direct impact on noise pollution.

In Table 2, the average noise levels during different stages are shown; however, these data do not convey the noise range between the higher and the lower measured values. Thus, more detail about the variability of noise levels regarding Stages A, B and C can be seen in Table 3. The LA90 shows a significant decrease (10 dB) between Stage A and Stage B in 2020. This means that noise levels were lower than in Stage A, resulting in a lower background noise level. Regarding this, LA10 also shows a significant decrease of around 5 dB. Consequently, the maximum noise levels to which the inhabitants of Barcelona were exposed during this period decreased significantly compared to Stage A of 2020, and the first semesters of 2019 and 2018. In addition, the average noise exposure in Stage B was also lower—see Table 2. In Stage C, the average noise levels increase again due to the de-escalation process. Accordingly, LA10 and LA90 levels increase during 2020. Even so, values are still lower than those in Stage A, and also than in Stages A, B and C for 2019 and 2018. Moreover, differences between the LA10–90 percentiles are observed in 2020 when Stage A is compared with Stage B and Stage C. On the one hand, during the more restrictive period (Stage B) *LA*10–90 shows a variation of over 6 dB (2020 to 2018–2019 column). On the other hand, during Stage C, LA10–90 shows a variation of 4.4 dB (2020 to 2018–2019 column). This is due to the significant reduction in LA90 levels during Stage B, and the subsequent significant increase in Stage C.The noise level difference (2020 to 2018–2019 column) between the lowest and the highest noise level (*LA*10–90) is negligible for Stage A. This behavior can be correlated with the mean values shown in Table 2 for the same stage. Regarding this, $L_{Aeq}$ for 2020, 2019 and 2018 show a minimum difference between them and are quite similar. It should be noted that Stage A is free from any COVID-19-related restriction.

#### 4.1.3. Global Daily Noise Indicators

**Figure 4:**
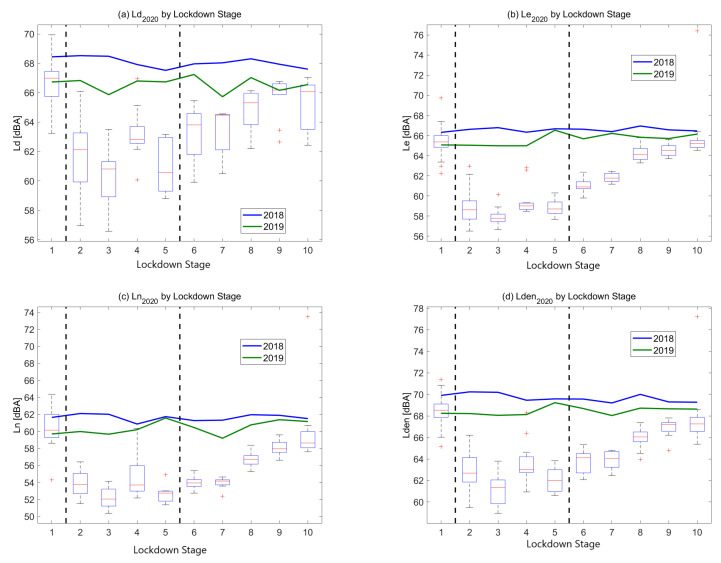
Global daily noise indicators in Barcelona during the different lockdown stages in 2020 compared to median 2018 and 2019 values. (**a**) Global $Ld_{2020}$. (**b**) Global $Le_{2020}$. (**c**) Global $Ln_{2020}$. (**d**) Global $Lden_{2020}$.

Figure 4 shows global daily noise indicators (i.e., Ld, Ln, Le, Lden) considering the ten different stages proposed. In addition, 2020’s daily indices are compared with the median values obtained during the same periods in 2018 and 2019. Ld is calculated as the daily average noise level from 7 to 21, Le from 21 to 23 and Ln from 23 to 7. Finally, Lden indicates the noise level over an entire day, imposing a penalty on the sound level during the evening and night periods.

To begin with, behavior of curves that show median noise levels in 2018 and 2019 are quite similar when compared for each daily noise index (i.e., Figure 3a Ld by 2018 and 2019 curves). Furthermore, curves are comparable for both years but the levels in 2019 are approximately 2 dB lower than in 2018. This trend is given in all figures (i.e., Figure 4a–d. Focusing on 2020, the noise levels obtained during the day period ($Ld_{2020}$) show a wide range of different values. During Stage 2 and 3, median noise levels are about 5 dB lower when compared with Stage 1. After that, mean levels start to rise gradually.

Looking at Le and Ln indicators in Figure 4, mean values are lower than Ld regardless of the stage. Specifically, the level measured in Stage 3 is the lowest for both indicators. In addition, the trend of the average noise levels through the stages is similar for Le and Ln. Noise values vary accordingly with the restrictions in Barcelona. Even so, Ln levels are about 6 dB lower than for Le irrespective of the stage.

Furthermore, the Lden indicator shows the same behavior as Le and Ln in all stages. Levels decrease until Stage 3 and gradually begin to rise from Stage 4 to Stage 10. However, they do not fully recover to pre-lockdown values, as in Stage 10 they remain approximately 1 dB lower than for the same period in 2019. Moreover, Lden levels from Stage 3 to Stage 10 go from $\tilde{6}$2 dBA to $\tilde{6}$7 dBA in 2020. For the same period in 2019, levels are about 68 dBA. In 2018, levels from Stage 3 to Stage 10 are quite constant and about 70 dBA.

In order to further study the correlation between the different restrictions adopted during the lockdown and their effects in the soundscape of Barcelona, a clustering algorithm was implemented on the main acoustic metrics of the ten stages. Specifically, the sound indices considered were the average LAeq, LA10, LA90, LA10–90, Ld, Le and Ln for each stage from Stage 1 to Stage 10 and the chosen algorithm was *k*-means.

An interesting outcome was revealed when the clustering was performed with *k* = 2. On the one hand, Stages 2 to 7 (corresponding to Stage B and the first weeks of Stage C) were grouped together. On the other hand, Stages 8 to 10 (second half of Stage C) were grouped with Stage 1 (Stage A). If we correlate this clustering with the standing regulations in each period, we find that the stages where shops, museums and sport centers were allowed to open were grouped with the strict lockdown stages. On the contrary, the opening of bars and restaurants in the first place and of entertainment venues and traffic outside the city later on, led to the same grouping as the normal pre-lockdown situation. This can shed some light on the effectiveness of possible policies to reduce background noise in the city. As the difference in grouping takes place with the opening of bars and restaurants, it may suggest that regulations limiting the noise in these activities or limiting the access to them by private transportation could have a bigger impact than policies affecting other activities.

#### 4.1.4. Global $L_{Aeq}$ by Day of the Week

**Figure 5:**
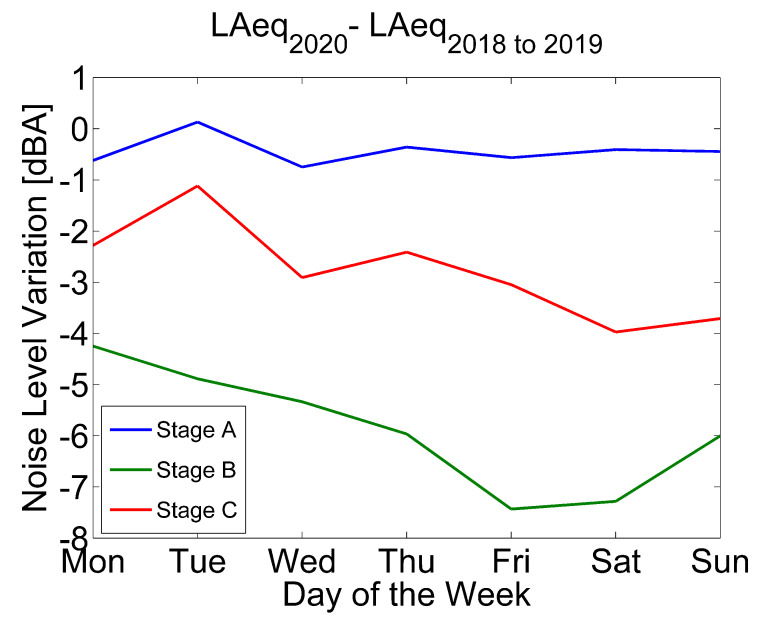
Noise reduction between the first semester of 2020 and the averaged equivalent periods of 2018 and 2019 by lockdown stage and day of the week.

Figure 5 presents the noise variation by days of the week during the first semester of the three years in study. In particular, it depicts the difference between 2020’s main lockdown Stages (i.e., A, B and C) and the averaged 2018–2019 equivalent time frames.

As was to be expected, noise levels in Stage A present no significant reduction in comparison with the average $L_{Aeq}$ from 2018 to 2019. On the contrary, during Stage B, noise level reduction is significant regardless of the day of the week considered. Even so, the highest variation is attained on Friday and Saturday with a noise level reduction of over 7 dB. In addition, the curve on Stage B presents a difference in noise level that increases steadily from Monday to Friday. Afterwards, a slight decrease begins from Saturday until it reaches Monday again.

Looking at Stage C levels, the reduction curve trend from Monday to Wednesday is almost identical to Stage A, but 2 dB lower. Otherwise, values from Thursday to Sunday present a significantly higher reduction reaching 4 dB on Saturday.

#### 4.1.5. Other Analysis of City Metabolism during the Lockdown

The exceptional situation caused by COVID-19 made human mobility a key virus-transmitting element. The most clear measure to minimize transmission was to reduce mobility to the point of implementing a home-based lockdown, resulting in the city widely ceasing activity, both in services and in production and industrial sectors, and also in terms of mobility, with the exception of essential services. In the technical report [28,29] the Barcelona City Council detail that, from the beginning of the lockdown to its end, the reduction of traffic was of −77%, and in the two weeks with harsher lockdown conditions, it reached a mean of −82%, in comparison with the former years. These data are quite significant for the vehicles going in or out of the city (via *Rondes*) when taking into account the high-density roads of the city. The number of travelers of public transport also follows that trend, but it made less of an less impact to the noise analysis conducted in this work.

There was a significant reduction in the number of people present in various locations [28], apart from residents, and few commuters compared to standard and almost no international travelers—approximately 1 Million people less than the same months of the previous year. Moreover, a reduction in economic activity produced a GNP decrease of 3.8% in the first trimester of the year, in comparison to the same period in 2019. In particular, the building sector experienced a significant decrease of over 7%. The energy consumption also decreased substantially, especially during the hard lockdown conditions [30] and did not reach regular values after the end of the lockdown. It presented peak reductions of −35% with mean values of −15%. Water consumption was also affected during the lockdown: an increase of around +10% at home during the hard lockdown and a decrease of more than a 50% in Barcelona City Council’s own data, between −45% and −48% in industrial values, and between −32% and −35% in community consumption. A clear impact of the fact that people spent all day at home, and most of the consumption reported by companies was also heavily decreased. Air quality also improved, as several stations deployed in Barcelona to measure air quality [28,29] during the hard lockdown reported around −46% of $NO_{2}$, a reduction of −16.5% of PM10 and an increase of the tropospheric ozone. The $NO_{2}$ average daily concentration reached minimum historical levels since before 2015. This significant reduction is deeply correlated with the abrupt decrease in the city mobility due to lockdown restrictions (see Graph 103 [28]).

Finally, and the measure closer to this work, is the global evaluation of the Barcelona Noise Monitoring Network during the lockdown. The conclusion of the average measured by the technicians of Barcelona City Council ([28]) during *only* the most severe lockdown is a reduction of −5.5 dBA in the mid-traffic roads, a decrease of −17.7 dBA due to leisure and restaurants noise in terraces, −12 dBA due to the reduction of the airport noise next to El Prat Airport and a special high reduction of noise in leisure areas from 22 and after, during the night. Some of the values stated in these studies—and that we use in the following sections to describe the variations of noise in certain locations of the city—are observed now and in the future to conclude whether they can point out future trends of people’s behavior (e.g., teleworking, the use of the bicycles in the city) [31].

### 4.2. Selected Locations for Representative Sounds in the City

In this subsection, we deepen the analysis by means of detailing the values for several representative sensors. We conducted an analysis for all 70 sensors that gathered data during the 2020 lockdown, and we chose up to eight locations, including: (i) heavy-traffic area, (ii) moderate-traffic area, (iii) low-traffic and residential area, (iv) daytime leisure and restaurant area, (v) nightlife area, (vi) Superblock and shopping area, (vii) industrial and services area and (viii) park area. Although we assume that other locations could also be representative of the main activity they host, or of several noise sources, these give us a global idea of the changes occurred in Barcelona during the 2020 lockdown.

All the eight locations are described by a figure, detailing the evolution of the noise level $L_{Aeq}$ for all the stages of the lockdown (from January to June 2020), the evolution of the noise level $L_{Aeq}$ for the same period during 2019, averaged with 2018 if available, also with the various stages highlighted to compare with the former figure. The third figure corresponds to a day over day comparison of the $L_{Aeq}$ between year 2020 (during the lockdown) and the same day in 2019, and finally, we have an aerial picture of the location of the sound sensor, which gives us more details about the surroundings of the place, the possible main noise sources and the principal human activities around the sensor.

Additionally, in the first six locations, an extra figure showing the average noise reduction during 2020 compared to the two prior years in all similar locations was added. These figures show the difference between the average noise level during the first semester of 2020 and the average noise level during the first semesters of 2018 and 2019, for a cluster of sensors placed in analogous locations. It also contains the same difference for Stages A, B and C. The aim of these figures is to examine the variability among sensors placed to gather the same kind of predominant noise source. It was not possible to include this figure for locations 7 and 8 as there were not enough sensors in Barcelona’s network placed in industrial and park areas beyond the ones already singled out.

Data from these selected locations were used to conduct a one-way ANOVA test [32] to check whether the data used in this research work were statistically relevant or not. For all eight locations, the F-factor, i.e., the output of the ANOVA test, between pre-lockdown data (throughout STAGE A) and lockdown and post-lockdown data (throughout STAGE B and C) was much higher than one. These outcomes describe two groups of data with a different mean, which lets us reject the null hypothesis and conclude that the data are statistically relevant.

#### 4.2.1. Location 1: Heavy-Traffic Area

The first location corresponds to *Mitre, 137*, a location in the urban district of Sarrià-Sant Gervasi that corresponds to a *Heavy-Traffic Area*.

**Figure 6:**
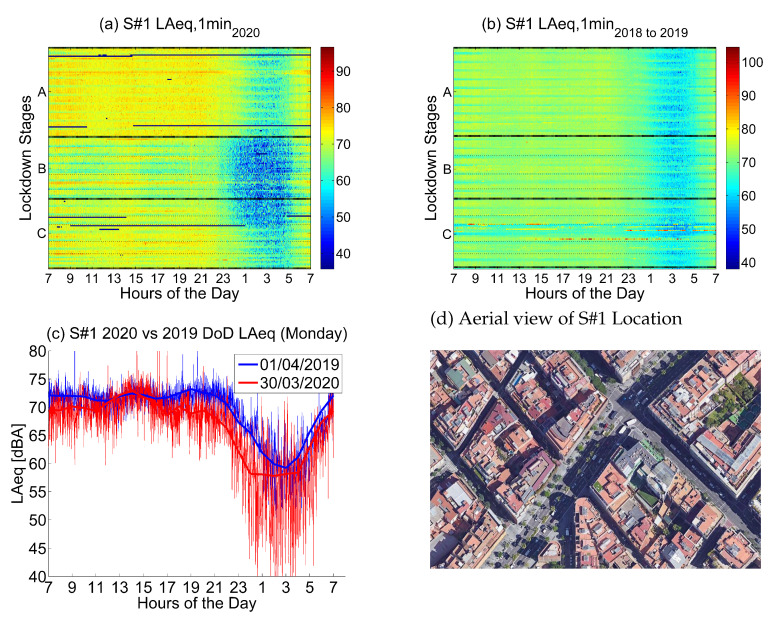
$L_{Aeq}$ variation in an area with heavy traffic (Mitre, 137): (**a**) $L_{Aeq},$1min${}_{2020}$. (**b**) $L_{Aeq},$1min${}_{2018to2019}$. (**c**) Day over day comparison of the hourly $L_{Aeq}$ trend between 2020 during the lockdown and 2019 on the same Monday. (**d**) Aerial photography.

Noise collected in the sensor in 2019 and 2018 (see Figure 6) presents high values during the day, especially from Monday to Friday, while Saturday and Sunday are quieter. Nights during the week are quieter from 23 to 6 in the morning; Thursday and Friday are exceptions.

Noise collected during the lockdown presents lower values during the day, but the same pattern of noise is kept. From Monday to Friday, the noise during the day is high—slightly lower than before the lockdown—a huge change is found during the night, especially the first stages of the lockdown (until end of May). Night values are clearly lower from 22 to 5 in the morning.

The day over day comparison in Figure 6c shows that even during the stage of tighter restrictions, noise levels in the morning and early afternoon fluctuated between a soft drop and even a slight increase at lunchtime compared to the same day in 2019. This unusual behavior was spotted on the main artery roads of the city center. In contrast, noise reduction was more evident from 17 on-wards.

The main noise source in this sensor comes from road traffic, so the first inference we can make is that most of the daily traffic is kept during the lockdown, probably due to the fact that it comes from an essential road in Barcelona, which most of the people crossing the city use. So, all essential services—health, food and other sectors—usually use that traffic artery for job purposes. Nevertheless, night traffic was clearly decreased during the lockdown.

**Figure 7:**
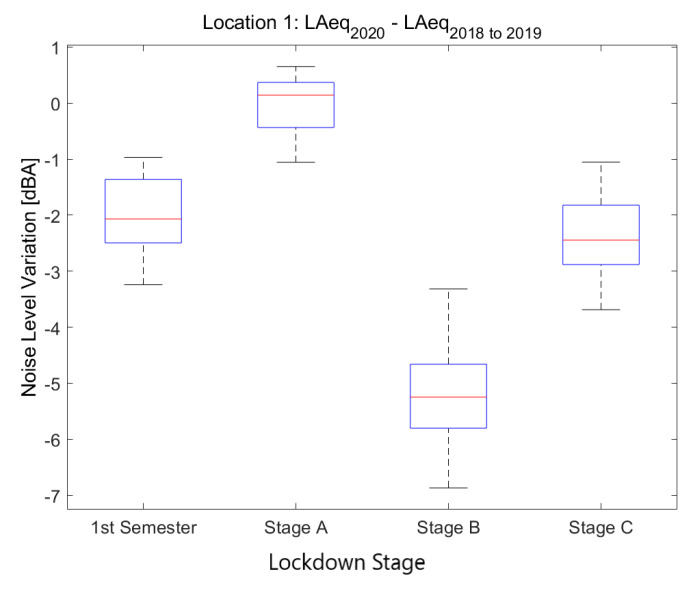
Average noise variation ($L_{Aeq},$1min${}_{2020}-L_{Aeq},$1min${}_{2018to2019}$) in all heavy-traffic sensor locations during the first semester and the three main lockdown stages of 2020 compared to the averaged same periods of 2018 and 2019.

Figure 7 shows a similar behavior in up to eleven sensors representing Heavy-Traffic Areas located in some of the main roads in the inner city. All these locations have an average $L_{Aeq},$ 1min ${}_{2018to2019}$ above 67.5 dB. Even though in all kinds of locations there is an appreciable increment of variance during Stages B and C compared to Stage A, this increment is distinctly lower in Heavy-Traffic Areas. Thus, the pattern described in *Mitre, 137* is truly representative of the sound variation in all thoroughfares of central Barcelona.

#### 4.2.2. Location 2: Moderate-Traffic Area

The second location corresponds to *Pg. Sant Joan, 39*, a location in the urban district of Eixample that corresponds to a *Moderate-Traffic Area*.

**Figure 8:**
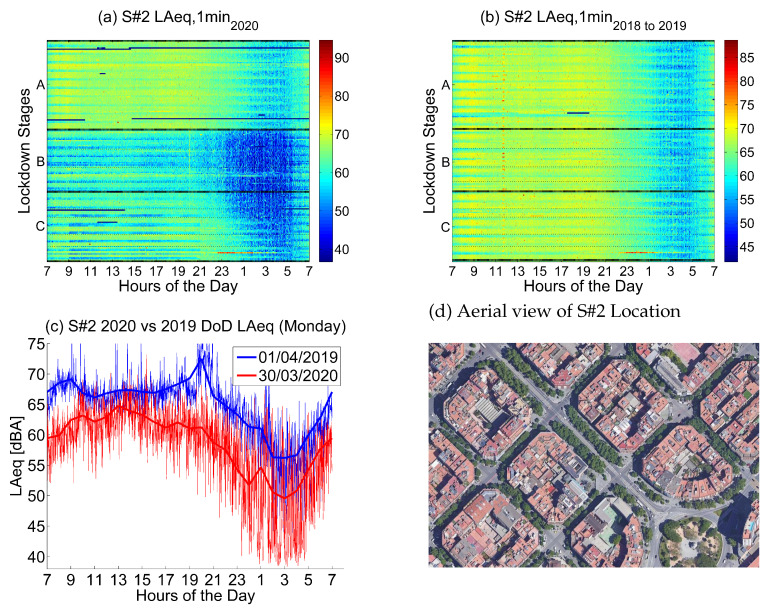
$L_{Aeq}$ variation in an area with moderate traffic (Pg. Sant Joan, 39): (**a**) $L_{Aeq},$1min${}_{2020}$. (**b**) $L_{Aeq},$1min${}_{2018to2019}$. (**c**) Day over day comparison of the hourly $L_{Aeq}$ trend between 2020 during the lockdown and 2019 on the same Monday. (**d**) Aerial photography.

Noise collected from this sensor in 2019 and 2018 (see Figure 8) presents high values during the day, especially from Monday to Friday; Saturday and Sunday are quieter. Nights during the week are quieter from 1 a.m. to 5 a.m. in the morning; Thursday and Friday are exceptions.

Noise collected during the lockdown presents lower values during the day, especially during the first two lockdown stages, until mid April. During the night, there is a huge decrease in noise levels, which is present until the first week of June. This decrease starts at 23 and finishes at 5 in the morning.

The main noise source in this sensor comes from road traffic, so the first inference we can make is that the daily traffic was decreased during the first two stages of the lockdown, but at that moment, it slowly recovered, despite not reaching the noise values previously measured in 2019 and 2018.

**Figure 9:**
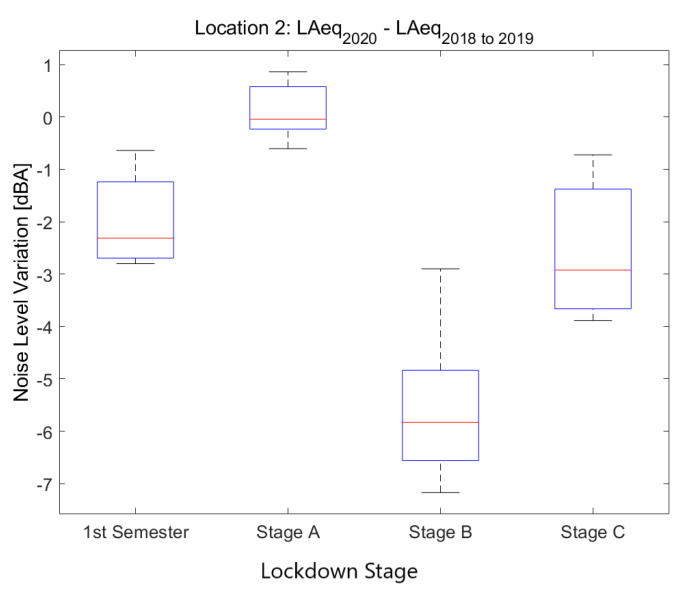
Average noise variation ($L_{Aeq},$1min${}_{2020}$ - $L_{Aeq},$1min${}_{2018to2019}$) in all moderate-traffic sensor locations during the first semester and the three main lockdown stages of 2020 compared to the averaged same periods of 2018 and 2019.

Figure 9 contains results coming from ten sensors located in moderate-traffic roads and arteries with an average $L_{Aeq},$1min${}_{2018to2019}$ level between 64.5 and 67.5 dB. Although the main source of noise in these locations is road traffic, there are also some shops, bars, pedestrian promenades and children’s playgrounds around. As expected, there is a higher variability among locations during the lockdown stages as opposed to the Heavy-Traffic Areas.

#### 4.2.3. Location 3: Low-Traffic Residential Area

The third location corresponds to *Torre dels Pardals, 96*, a location in the urban district of Horta-Guinardó, which is a *Low-Traffic and Residential Area*.

**Figure 10:**
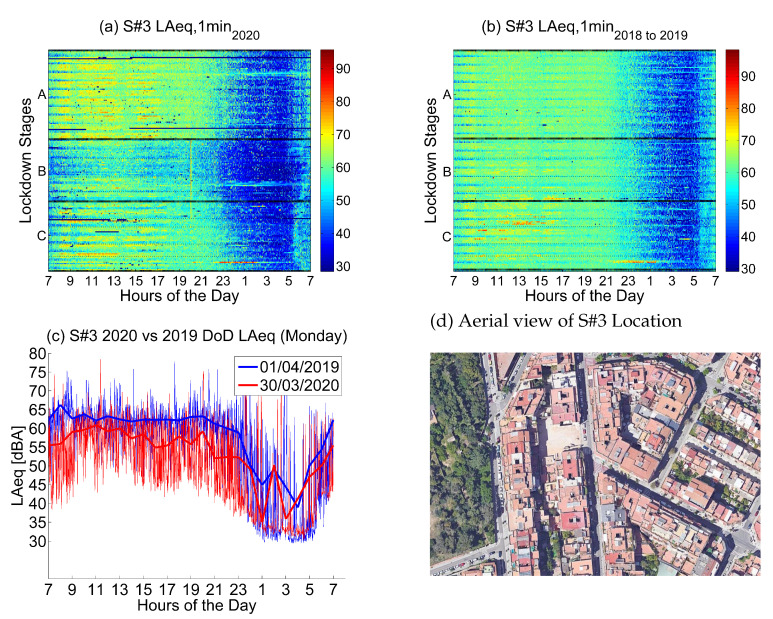
$L_{Aeq}$ variation in a residential area with low traffic (Torre dels Pardals, 96): (**a**) $L_{Aeq},$1min${}_{2020}$. (**b**) $L_{Aeq},$1min${}_{2018to2019}$. (**c**) Day over day comparison of the hourly $L_{Aeq}$ trend between 2020 during the lockdown and 2019 on the same Monday. (**d**) Aerial photography.

Noise collected from this sensor in 2019 and 2018 (see Figure 10) presents moderate values during the day, especially from Monday to Friday; Saturday and Sunday are quieter. Nights during the week are quieter from 23 to 5 in the morning.

Noise collected during the lockdown presents lower values during the day, especially during the first two lockdown stages, until mid April. During the night, there is a huge decrease in the noise levels, clearly visible until the first week of June. This decrease starts at 22 and finishes at 5.

One of the noise sources in this place comes from traffic, which was clearly reduced during the first part of the lockdown. In addition, the traffic noise in this area comes from the mobility of neighbors, as it is a residential area. The activity in the neighborhood was slowly recovered from that moment on, presenting lower noise values but higher than in the previous stages of the lockdown.

**Figure 11:**
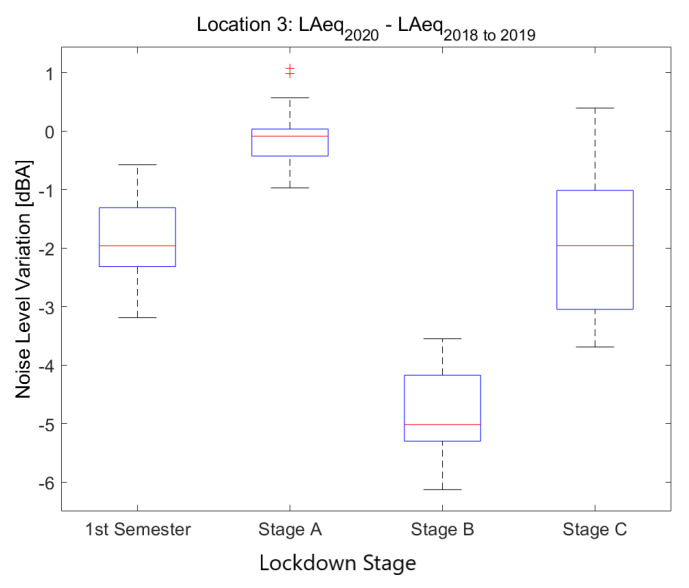
Average noise variation ($L_{Aeq},$1min${}_{2020}$ - $L_{Aeq},$1min${}_{2018to2019}$) in all low-traffic residential sensor locations during the first semester and the three main lockdown stages of 2020 compared to the averaged same periods of 2018 and 2019.

In Figure 11, 14 low-traffic areas are considered. These sensors are located in quieter streets, mostly surrounded by residential areas in different districts of the city. All of them have an average $L_{Aeq},$1min${}_{2018to2019}$ level below 64.5 dB. Compared to Figure 9, there is a higher variance during the final stages of lockdown (corresponding to the de-escalation plan). There are three main reasons that can explain this disparity. Firstly, not all residential areas have the same services in the neighborhood and some of them are more densely populated than others. Secondly, during the lockdown stages, some secondary streets had their road traffic completely removed and some of them were temporarily transformed to pedestrian walkways when the home lockdown was relaxed. These measures were unevenly reverted during the final stages, resulting in a wider difference in the noise soundscape for these residential areas. Finally, teleworking has also unequally reduced mobility in these residential neighborhoods. The impact of teleworking and temporary moving to second homes has been significantly higher in high-income districts as opposed to the poorer ones.

#### 4.2.4. Location 4: Daytime Leisure and Restaurants Area

The fourth location corresponds to *Plaça Eivissa, 21*, a location in the urban district of Horta-Guinardó that is consistent with a *Daytime leisure and Restaurants Area*.

**Figure 12:**
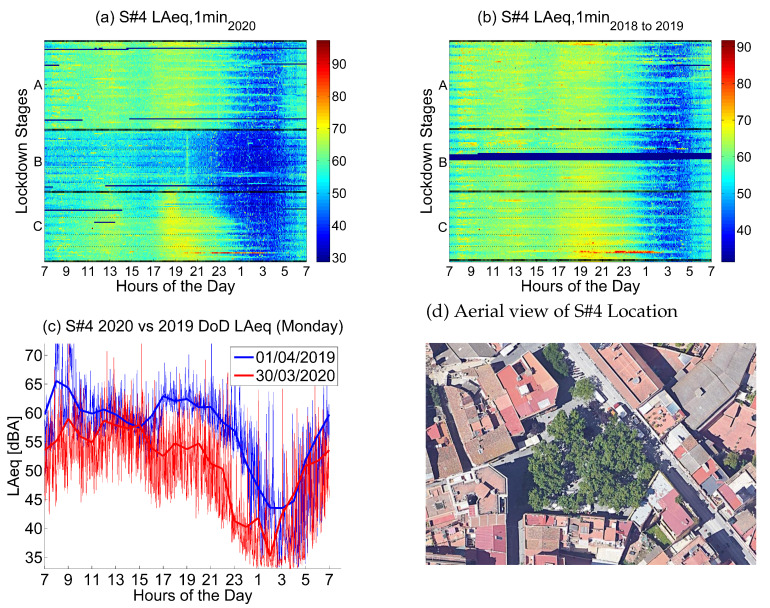
$L_{Aeq}$ variation in a bars and restaurants area (Pl. Eivissa, 21): (**a**) $L_{Aeq},$1min${}_{2020}$. (**b**) $L_{Aeq},$1min${}_{2018to2019}$. (**c**) Day over day comparison of the hourly $L_{Aeq}$ trend between 2020 during the lockdown and 2019 on the same Monday. (**d**) Aerial photography.

Noise collected in that sensor in 2019 and 2018 (see Figure 12) presents moderate values during the day, with several waves corresponding to the morning—school and jobs begin—between 8 and 9, and the afternoon—school and jobs end—together with commerce and even early dinner—between 17 and 21, especially from Monday to Friday, stating that Saturday and Sunday are quieter, and following later patterns. Nights during the week are quieter from24 to 5 in the morning.

Noise collected during the lockdown presents lower values during the day, especially during the first three lockdown stages, until the end of April. During the night, there is a huge drop in the noise levels, clearly visible until the first week of June. This decrease starts at 22 and finishes at 6 am, being longer in the morning during the weekends. Moreover, during Stage B, this steep dip is also evident during afternoons as exemplified in Figure 12c. As this location is filled with restaurants and cafeterias, the dinner time frame from 17 to midnight experienced an additional fall in the noise level during Stages 2 to 5.

The noise sources in this location come from traffic and outdoor life—people in the streets, commerce and restaurants—which was clearly decreased during the first part of lockdown. The activity in the neighborhood slowly recovered from the third stage of the lockdown onward, presenting still lower noise values but higher than in the previous stages of the lockdown.

**Figure 13:**
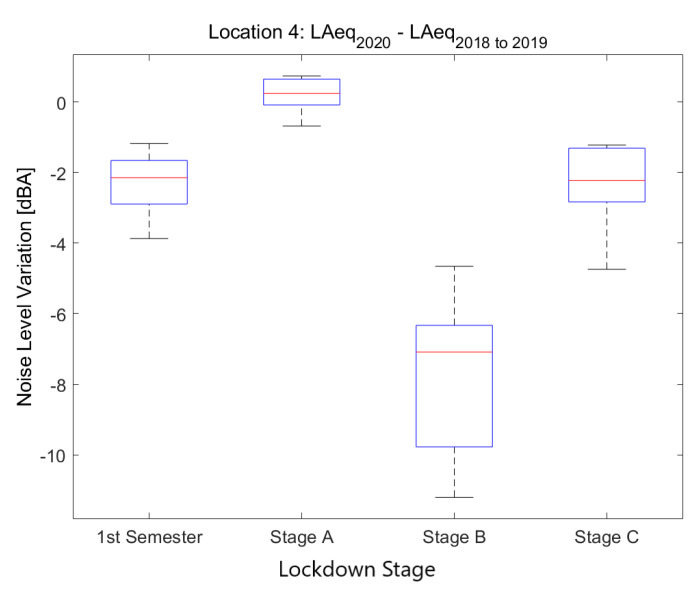
Average noise variation ($L_{Aeq},$1min${}_{2020}$ - $L_{Aeq},$1min${}_{2018to2019}$) in all sensor locations dedicated to daytime leisure during the first semester and the three main lockdown stages of 2020 compared to the averaged same periods of 2018 and 2019.

Figure 13 compares all available locations in areas dedicated to daytime leisure. These areas are mainly town squares filled with restaurants, bars, cafeterias and ice-cream shops. In most of them, road traffic is limited or nonexistent. Therefore, the main source of noise comes from people talking and shouting, dogs and other pedestrian and restaurant-related sounds. In addition to that, there is also some commerce activity in the same spots. However, these squares are located in the middle of residential districts. There is some road traffic unevenly present in the surrounding area. Furthermore, in some of the locations there is also an overlapping with some nightlife activity, especially between 23 and 2.

A significantly higher variability among sensors during Stage B is observed, especially when compared to the final stages of lockdown. This was to be expected because restaurants and bars were where all the noise originated from, and were bound to present a deeper plunge than those with some road traffic in the neighboring streets. This makes it more difficult to generalize the results in Figure 12 to all daytime leisure locations during the peak of the lockdown.

#### 4.2.5. Location 5: Nightlife Area

The fifth location corresponds to *Escudellers, 48*, a location in the urban district of Ciutat Vella, which is a *Nightlife Area*.

**Figure 14:**
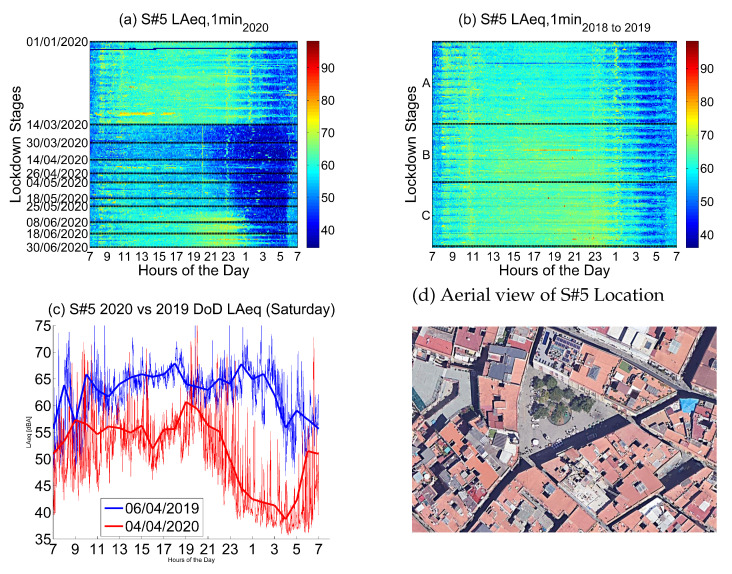
$L_{Aeq}$ variation in a nightlife area (Escudellers, 48): (**a**) $L_{Aeq},$1min${}_{2020}$. (**b**) $L_{Aeq},$1min${}_{2018to2019}$. (**c**) Day over day comparison of the hourly $L_{Aeq}$ trend between 2020 during the lockdown and 2019 on the same Saturday. (**d**) Aerial photography.

Noise collected from this sensor in 2019 and 2018 (see Figure 14) presents moderate values during the day, with irregular patterns coming mainly from the street life, but with a clear wave starting in the afternoon—around 17—which finishes between 2 and 3 in the morning, from Monday to Friday, and even later—up to 5—during the weekends. The main activity is leisure and nightlife noise, leaving the quiet period of the night from 3 to 6 in the morning during the week and, during the weekend, the figure states that there is nearly no silent part of the night at the street level.

Noise collected during the lockdown presents lower values during the day, especially during the first two lockdown stages, until the end of April. During the night, there is a huge decrease in the noise levels, clearly visible until the first week of June. This decrease starts at 21 and finishes at 6, which is a very relevant change in comparison with the previous measurements. In this sense, nights keep being slightly quieter even in the end of June, probably due to the still valid restrictions for night leisure.

The main noise sources in this place come from nightlife and also from commerce and outdoor tourist activity, which were clearly decreased during the first part of lockdown. The activity in the neighborhood slowly recovered from the third stage of the lockdown onward, basically during the day, presenting still lower noise values but higher than in the previous stages of the lockdown. The values collected were larger by the end of June, not reaching the former measurements in 2019 and 2018, but obtaining higher values from 19 to 24 nearly every day, and until 3 in the weekends.

**Figure 15:**
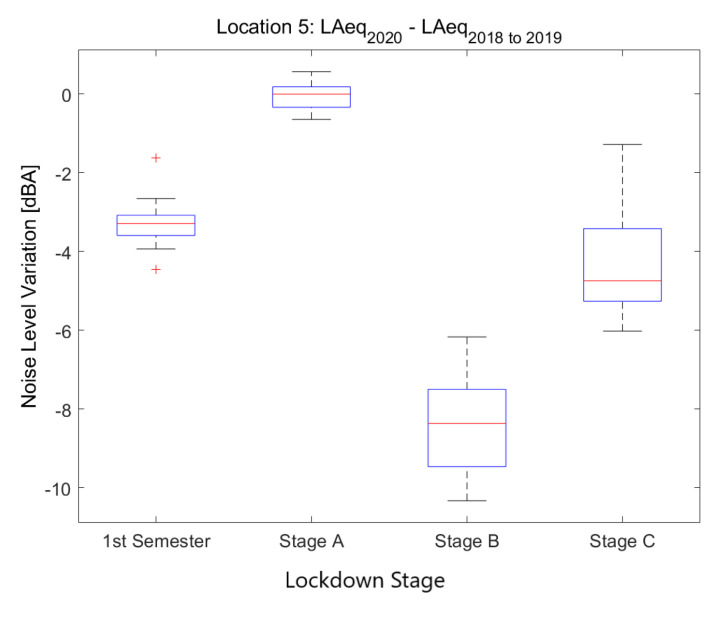
Average noise variation ($L_{Aeq},$1min${}_{2020}$ - $L_{Aeq},$1min${}_{2018to2019}$) in all sensor locations dedicated to nightlife during the first semester and the three main lockdown stages of 2020 compared to the averaged same periods of 2018 and 2019.

In Figure 15, the nine sensors deployed to collect nightlife soundscapes are studied. Six of them are located in the district of Ciutat Vella, which is mostly filled with pedestrian streets, pubs, clubs and tourist attractions that, on the whole, have displaced most local residents to other districts. Therefore, most of the noise is related to nightlife life, commerce and tourism. The other three sensors are placed in three other districts in areas where nightlife is predominant too. As expected, the average $L_{Aeq}$ plummeted during the lockdown in all nightlife areas. However, the noise level decrease was more severe in some spots than others according to the presence or absence of other secondary sources of noise in the proximity of the sensor.

#### 4.2.6. Location 6: Superblock and Shopping Area

The sixth location corresponds to *Rda. St. Antoni, 59*, a location in the urban district of Ciutat Vella that corresponds to a *Superblock and Shopping Area*.

**Figure 16:**
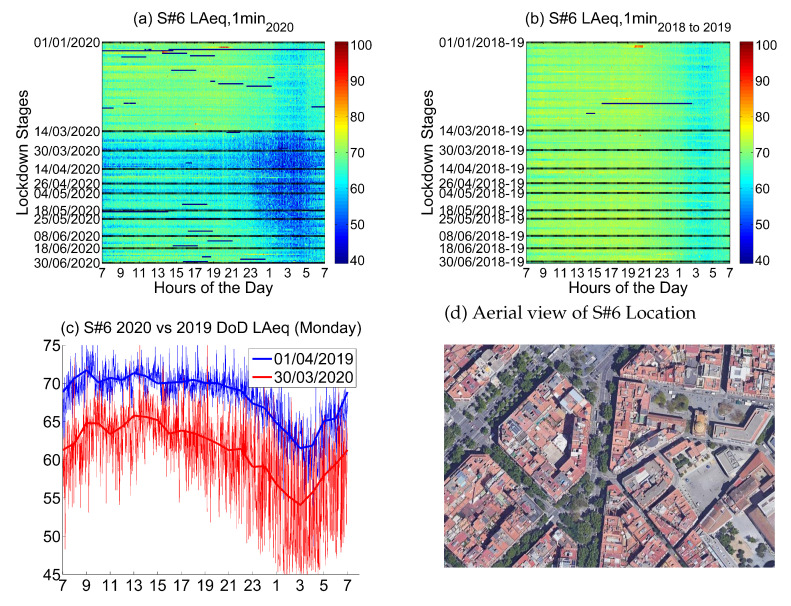
$L_{Aeq}$ variation in a Superblock and shopping area (Rda. St. Antoni, 59): (**a**) $L_{Aeq},$1min${}_{2020}$. (**b**) $L_{Aeq},$1min${}_{2018to2019}$. (**c**) Day over day comparison of the hourly $L_{Aeq}$ trend between 2020 during the lockdown and 2019 on the same Monday. (**d**) Aerial photography.

Noise collected in that sensor in 2019 and 2018 (see Figure 16) presents moderate values during the day, with irregular patterns coming mainly from the street life, but with a clear raise starting around 9, which starts to decrease just after lunch—around 15—reaching the minimum at 3 in the morning. The performance is not the same during the weekday than during the weekend, when the nights have higher noise and the days and slightly more moderate values, probably due to less traffic.

Noise collected during the lockdown presents lower values during the day, especially during the first two lockdown stages, until the end of April. During the night, there is a huge decrease in the noise levels, clearly visible until the first week of June. This decrease starts at 23 and finishes around 6, which is a very relevant change in comparison with the previous measurements. Even so, noise reduction in this location during Stage B was quite homogeneous throughout the day as seen in Figure 16c. Although activity in the Superblock has more or less recovered in the street during June, the noise measured falls short of the former values collected in 2019 and 2018.

The main noise sources in this place come from outdoor life, restaurants, commerce and nightlife; all these activities were mainly stopped during the first part of lockdown. The activity in the neighborhood was slowly recovered from the third stage of the lockdown onward, basically during the day.

**Figure 17:**
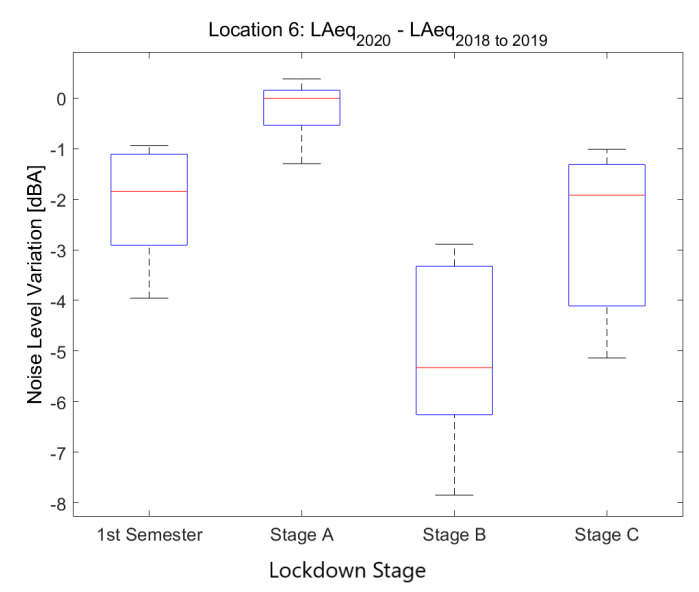
Average noise variation ($L_{Aeq},$1min${}_{2020}$ - $L_{Aeq},$1min${}_{2018to2019}$) in all sensor located in Barcelona’s Superblocks during the first semester and the three main lockdown stages of 2020 compared to the averaged same periods of 2018 and 2019.

The goal of Barcelona’s Superblock project is to reduce the road traffic in residential areas, especially traffic derived from private vehicles. The project also aims to create greener and healthier public space. That being said, not all Superblocks are in the same stage of transformation; therefore, the presence of road traffic is unequal. Furthermore, the exact location of sensors have a lot of influence on the results. Those located near the boundaries of the Superblock are more prone to collect road traffic noise than the ones located in the inner streets. Type of activity inside the Superblock has relevance too. The presence of shops, restaurants, parks or schools lead to different soundscapes. Comparing data from sensors located in five different Superblocks (see Figure 17), a significant variability of the noise reduction during all the lockdown stages is spotted. Thus, it would be wrong to assume that the chosen example in Figure 16 is an accurate depiction of 2020’s noise pattern in all Superblocks across Barcelona.

#### 4.2.7. Location 7: Industrial and Services Area

The seventh location corresponds to *Ulldecona, 77*, a location in the industrial part of the district of Sants-Montjuïc, which is an *Industrial and Services Area*. There is a gas station and several parking lots near the sensor.

**Figure 18:**
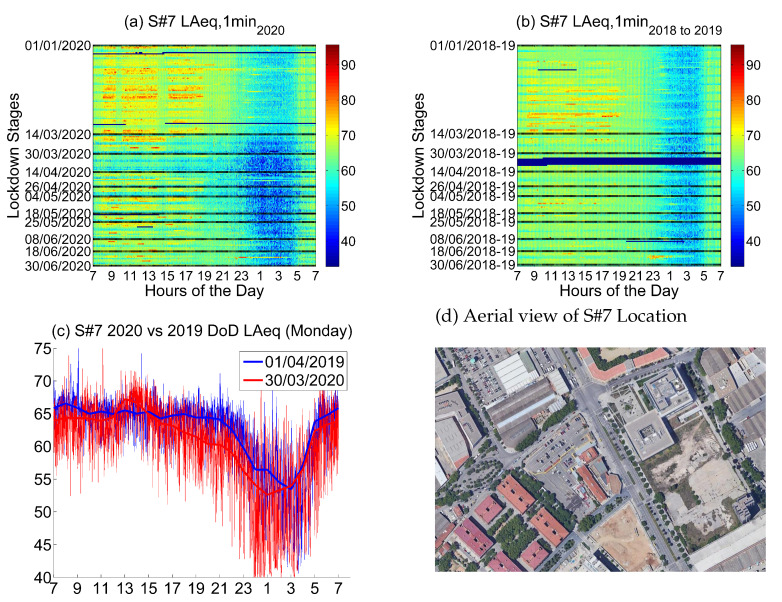
$L_{Aeq}$ variation in an industry and services area (Ulldecona, 77): (**a**) $L_{Aeq},$1min${}_{2020}$. (**b**) $L_{Aeq},$1min${}_{2018to2019}$. (**c**) Day over day comparison of the hourly $L_{Aeq}$ trend between 2020 during the lockdown and 2019 on the same Monday. (**d**) Aerial photography.

Noise collected from this sensor in 2019 and 2018 (see Figure 18) presents high values during the day, especially from Monday to Friday; Saturday and Sunday are quieter, which points out that is an industrial area with more activity in the weekdays. Nights are usually quiet 23 to 5 in the morning, and in that sense, the weekends do not present clear changes.

Noise collected during the lockdown presents lower values only during the second stage, not the first one or even the third one. For most of the stages of the lockdown, from Monday to Friday, the noise during the day is high and a clear change is found is during the night, especially for the first stages of the lockdown (until end of May). Night values are clearly lower from 23 to 5 in the morning.

The main noise source in this sensor comes from industry and road traffic, so the first inference we can make is that most of the daily traffic is kept during the lockdown, as well as most of the industrial activity.

There is an apparent anomaly in the day to day comparison in Figure 18c. The local maximum around 13:30 suggests a change of habits from the workers in this area. During the lockdown, non-essential services in this non-residential neighborhood were closed. Therefore, the main road traffic was caused by essential workers coming or going to their houses. Normally, they would be able to have lunch in a restaurant nearby and return home during the afternoon. At the end of March, that was not possible. Furthermore, a large part of essential workers in the area work mainly in the morning shift, e.g., delivery suppliers. Thus, most workers returned early home at lunchtime.

#### 4.2.8. Location 8: Park Area

The eighth location corresponds to *Eiximenis, 21*, a location in the urban part of the district of Sant Andreu, which is a *Park Area*.

**Figure 19:**
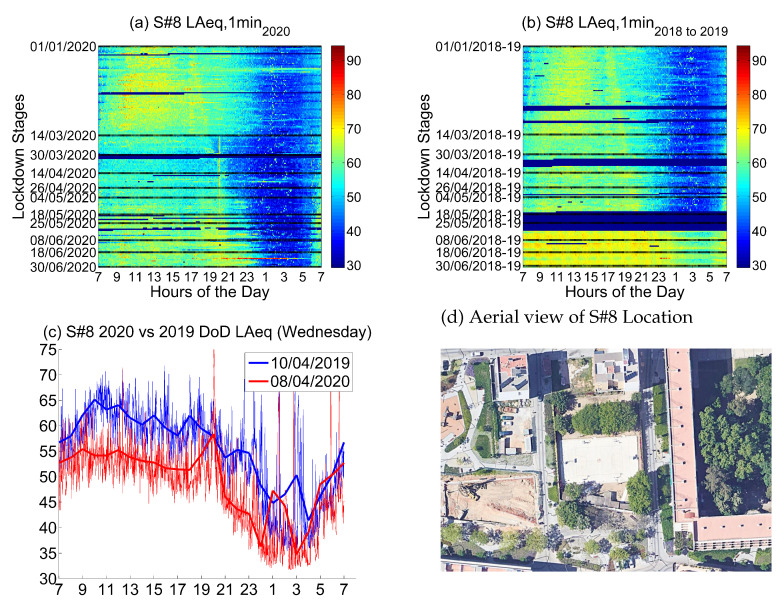
$L_{Aeq}$ variation in a park area (Eiximenis, 21): (**a**) $L_{Aeq},$1min${}_{2020}$. (**b**) $L_{Aeq},$1min${}_{2018to2019}$. (**c**) Day over day comparison of the hourly $L_{Aeq}$ trend between 2020 during the lockdown and 2019 on the same Wednesday. (**d**) Aerial photography.

Noise collected from this sensor in 2019 and 2018 (see Figure 19) presents usual low values during the night—with short exceptions in the measurements, probably due to short conversations in the street at night—and medium values during the day. There is a clear trend showing that the strict winter time the noise starts later in the morning (11) and finishes earlier in the afternoon (17), while later in spring, the noise starts much earlier in the morning around 9, and finishes around 20 in the evening. These values are much higher during the summertime, where even the night silence is nearly negligible—in winter, low values from 21 to around 7 are found.

In this location, it is very important to compare week to week, due to the fact just stated. The behavior of the people developing activities outdoor is very different depending on the month of the year. Nevertheless, there is a decrease in noise during the two first stages of the lockdown, with a slight increase of noise during the third and the fourth stage. The following stages show that the activity is back to the urban park, but the nights keep silent, with a clear lower noise values than before the confinement for the same weeks.

The main noise source in this sensor comes from outdoor activities of people in the park. These activities were forbidden during the most strict stages of the lockdown, and allowed with limitations in the following stages, as the figure states. When outdoor life is widely allowed, close to the end of spring and the beginning of summer, the values of noise raise again, despite not reaching the former values before the lockdown.

#### 4.2.9. Daily Noise Indicators in the Selected Sensors

In this subsection, a comparison of the average noise reduction during the day, evening and night periods is performed for the eight chosen locations and the three main lockdown stages.

If we consider the stricter stages of lockdown, corresponding to Stage B in Table 4, the locations with a higher noise reduction between 7 and 19 h periodare the roads with moderate-traffic noise and the areas with a predominant presence of commerce, nightlife or restaurants. On the contrary, the city’s main arteries and industrial zones were less affected by the restrictions. During the final stages of the lockdown, the same tendency was observed with two noticeable exceptions. First, daytime leisure recovered much faster than nightlife. Second, parks continued to be even quieter during the last months.

With the exception of parks and daytime leisure, noise reduction during the evening and night followed similar patterns. As was to be expected, the single location that experienced a sharper nightly drop during all the lockdown stages was the one related to nightlife. Commercial and moderate-traffic areas also experienced a substantial fall in noise levels during the night. On the other hand, main roads, residential and industrial areas presented lower variations at evening and night during the lockdown.

Daytime leisure and restaurants areas present a harsher drop during evenings than at nights. This comes as no surprise given that from 9 p.m. to 11 p.m. Barcelona residents are usually having dinner and there is an increment of activity in restaurants.

Surprisingly, the single sensor that registered a heavier fall in $L_{n}$ during the final stages of de-escalation is the one located in a park. During Stage B, the decrease at evenings was clearly higher than during Stage C. That was to be expected since people were locked inside their homes during Stage B. However, at night, there is a higher than expected fall in the noise level during Stage C. That is due to an abnormally high $L_{Aeq}$ in this park during May and June of 2019. For that reason, levels during Stage C for location 8 may not be representative of the noise reduction pattern caused by the lockdown.

## 5. Conclusions

Barcelona experienced a significant drop in $L_{Aeq}$ during the lockdown stages in 2020 (Figure 3 consistent with other research conducted in several cities around the world, such as Dublin [10], Buenos Aires [9], Madrid [11] and Milan [12]. The decrease was specially steep during the stages with stricter mobility and activity restrictions, i.e., Stage B, and during the night hours. This decline has to be put in context as Barcelona was already showing a mild but steady noise reduction trend in most of its sensors from 2018 to the first months of 2020, probably due to the pacifying efforts being implemented in recent years by the local administration.

Percentiles also show a higher variability in noise levels during the lockdown. Noise reduction is generally correlated with stricter restrictions in all the analyzed areas and in Barcelona’s global trends. Recovery to pre-lockdown noise levels was not yet attained by the end of June in most of the locations due to remaining mild restrictions during the last de-escalation phase and the change of habits caused by the pandemic. The recovery pace during Stage C was uneven depending on the main source of noise. Road traffic increased much faster than nightlife and tourism-related noise, as was to be expected because the international mobility was severely affected even during the final stages of the lockdown.

Intraweek noise reduction during the first semester of 2020 compared to the 2018–2019 period changed during the lockdown stages. While during Stage B the highest decrease took place during Friday and Saturday, it shifted to Saturday and Sunday during the de-escalation process. This can be explained by the opening of non-essential activities such as restaurants and commerce combined with the flow of citizens leaving the city during the weekend when the perimeter lockdown was lifted.

Even though all areas produced lower $L_{Aeq}$ levels during the lockdown stages, they were not equally affected. Areas with heavy traffic experienced lower noise reduction than areas with moderate or low traffic, especially during the day.

As for the other sources of noise studied, they also showed differences. On the one hand, daytime leisure and restaurant areas and nightlife areas were among the most affected, with distinct intraday noise variation. Nightlife areas took a huge plunge during the evening and night time frames, whereas daytime leisure and restaurant areas were most affected during afternoons and evenings. Furthermore, Superblocks and shopping areas presented a similar drop irrespective of the hour of the day. On the other hand, industrial and services areas were among the less affected by the restrictions, especially during the morning hours.

As opposed to most of the research published studying the noise variation during the lockdown in other cities, the lofty number of sensors available in Barcelona made it possible to analyze the representativeness of the chosen locations.

The higher the road traffic present on a specific location, the less variability among different sensors. Thus, a similar pattern was found in all the city’s arteries. On the contrary, there was a higher variability in moderate and, especially, in low traffic areas, where road traffic was not necessarily the only noise source. Locations with more than one predominant source of noise, i.e., Superblocks and shopping areas, were the most variant, making it difficult to conclude that the examples studied are representative of other similar areas in the city. Further, there were not enough sensors located in parks and industrial and service areas to draw conclusions about the representativeness of the examples depicted.

Finally, 2020’s restrictions caused by COVID-19 pandemic proved that the environmental noise of the city can be effectively decreased by implementing stricter mobility regulations and policies.

## Figures and Tables

**Table 1:** Summary of noise indices in Barcelona during the first semester of 2018, 2019 and 2020 and their variation compared to prior years.

Index	2020	2019	2018	2020 to 2019	2019 to 2018	2020 to 2018–2019
$L_{Aeq}$	63.1	64.8	66.2	−1.8 dB (−18.66%)	−1.4 dB (−14.37%)	−2.5 dB (−24.96%)
LA5	71.5	72.3	73.0	−0.8 dB (−8.8%)	−0.7 dB (−7.74%)	−1.16 dB (−12.47%)
LA10	69.6	70.7	71.5	−1.1 dB (−11.9%)	−0.8 dB (−8.8%)	−1.5 dB (−15.95%)
LA50	60.6	63.1	64.6	−2.5 dB (−25.01%)	−1.5 dB (−15.86%)	−3.3 dB (−31.47%)
LA90	45.7	50.6	53.46	−4.9 dB (−43.11%)	−2.9 dB (−28.06%)	−6.5 dB (−52.4%)
LA95	40.9	45.7	48.5	−4.8 dB (−42.46%)	−2.8 dB (−27.56%)	−6.3 dB (−51.65%)
LA10–90	23.9	20.1	18.04	3.8 dB	2.1 dB	4.9 dB

**Table 2:** The $L_{Aeq}$ in Barcelona during the first semester of 2018, 2019 and 2020 by lockdown stage.

Stage	2020	2019	2018	2020 to 2019	2019 to 2018	2020 to 2018–2019
Stage 1	65.1	64.7	66.4	0.4 dB (4.26%)	−1.7 dB (−17.66%)	−0.5 dB (−5.83%)
**Stage A**	**65.1**	**64.7**	**66.4**	**0.4 dB (4.26%)**	**−1.7 dB (−17.66%)**	**−0.5 dB (−5.83%)**
Stage 2	59.7	64.7	66.7	−4.9 dB (−43.24%)	−2.0 dB (−20.61%)	−6.0 dB (−49.76%)
Stage 3	58.2	64.6	66.2	−6.3 dB (−51.76%)	−1.6 dB (−17.09%)	−7.2 dB (−56.27%)
Stage 4	60.8	64.3	65.8	−3.5 dB (−33.0%)	−1.5 dB (−16.1%)	−4.3 dB (−38.87%)
Stage 5	59.2	65.5	65.7	−6.3 dB (−51.82%)	−0.2 dB (−2.11%)	−6.4 dB (−52.33%)
**Stage B**	**59.5**	**64.7**	**66.2**	**−5.2 dB (−44.82%)**	**−1.5 dB (−15.84%)**	**−5.9 dB (−49.56%)**
Stage 6	61.1	65.2	66.0	−4.1 dB (−37.82%)	−0.8 dB (−9.07%)	−4.6 dB (−40.77%)
Stage 7	61.2	65.8	65.5	−4.7 dB (−41.52%)	0.3 dB (3.32%)	−4.5 dB (−40.56%)
Stage 8	62.8	65.1	66.2	−2.2 dB (−22.66%)	−1.1 dB (−11.98%)	−2.8 dB (−27.59%)
Stage 9	63.8	64.8	65.6	−1.0 dB (−11.09%)	−0.9 dB (−9.32%)	−1.5 dB (−15.44%)
Stage 10	64.4	65.3	66.2	−1 dB (−10.46%)	−0.9 dB (−9.86%)	−1.4 dB (−15.1%)
**Stage C**	**62.8**	**65.2**	**66**	**−2.4 dB (−23.73%)**	**−0.8 dB (−9.07%)**	**−2.8 dB (−27.35%)**

**Table 3:** The LA10–90 in Barcelona during the first semester of 2018, 2019 and 2020 by lockdown stage.

Stage	Index	2020	2019	2018	2020 to 2019	2019 to 2018	2020 to 2018–2019
	LA10	70.9	70.7	71.7	0.2 dB	−1.0 dB	−0.3 dB
Stage A	*LA*90	51.3	50.4	53.5	0.9 dB	−3.1 dB	−0.8 dB
	LA10–90	19.6	20.3	18.2	−0.7 dB	2.1 dB	0.5 dB
	LA10	66.5	70.5	71.4	−4.0 dB	−0.9 dB	−4.5 dB
Stage B	*LA*90	41.3	50.5	53.5	−9.2 dB	−3.0 dB	−10.83 dB
	LA10–90	25.2	20.0	17.9	5.2 dB	2.1 dB	6.4 dB
	LA10	69.1	70.8	71.4	−1.7 dB	−0.6 dB	−2.01 dB
Stage C	*LA*90	45.8	51.0	53.3	−5.2 dB	−2.3 dB	−6.43 dB
	LA10–90	23.3	19.8	18.1	3.5 dB	1.7 dB	4.4 dB

**Table 4:** Daily noise indicator variation in dB between 2020 and the 2018 to 2019 in the selected sensors by lockdown stage.

Index	Stage	S#1	S#2	S#3	S#4	S#5	S#6	S#7	S#8
	Stage A	0.4	−0.3	1.4	0.4	0.1	−1.4	2.2	1.3
Ld	Stage B	−2.9	−6.3	−3.9	−6	−7.7	−7.4	−0.8	−4.6
	Stage C	−0.7	−4	−1.3	−0.8	−3.3	−4.7	1.1	−5.0
	Stage A	0.4	−0	−0.1	1.7	1.1	−1.3	1.4	2.5
Le	Stage B	−4.3	−7.3	−5.1	−9.3	−12.1	−8.5	−3.7	−7.3
	Stage C	−1.3	−2.7	−1.64	−1.9	−2.9	−5	−1.2	−5
	Stage A	0.4	0.1	-0.1	1.2	0.0	−1	1.8	1.5
Ln	Stage B	−4.7	−7.5	−5.4	−6.5	−12.1	−9	−1.9	−1.5
	Stage C	−2.3	−4.2	−1.3	−2.5	−6.4	−6.6	−1.2	−8.6

## Abbreviations

The following abbreviations are used in this manuscript:

WHO	World Health Organization
EU	European Union
SPL	Sound Pressure Level
$L_{Aeq}$	A-weighted equivalent sound level
LAN	A-weighted Sound Pressure Level exceeded for N percent of the time
LAF	A-weighted Sound Pressure Level measured with a fast time-constant
Ld	Day Noise Level. A-weighted Leq, over the 14-h day period (7 to 21)
Le	Evening Noise Level. A-weighted Leq, over the 2-h evening period (21 to 23)
Ln	Night Noise Level. A-weighted Leq, over the 8-h night period (23 to 7)
$L_{den}$	Day–evening–night noise level. A-weighted Leq, over a whole day, but with a penalty
	of 10 dBA for nighttime noise (23 to 7) and 5 dBA for evening noise (21 to 23)
